# Estimated Health and Economic Outcomes of Racial and Ethnic Tuberculosis Disparities in US-Born Persons

**DOI:** 10.1001/jamanetworkopen.2024.31988

**Published:** 2024-09-10

**Authors:** Nicole A. Swartwood, Yunfei Li, Mathilda Regan, Suzanne M. Marks, Terrika Barham, Garrett R. Beeler Asay, Ted Cohen, Andrew N. Hill, Charles R. Horsburgh, Awal D. Khan, Donna Hubbard McCree, Ranell L. Myles, Joshua A. Salomon, Julie L. Self, Nicolas A. Menzies

**Affiliations:** 1 Harvard T.H. Chan School of Public Health, Boston, Massachusetts; 2 Division of Tuberculosis Elimination, US Centers for Disease Control and Prevention, Atlanta, Georgia; 3 Office of Health Equity, National Center for HIV, Viral Hepatitis, STD, and TB Prevention, US Centers for Disease Control and Prevention, Atlanta, Georgia; 4 Yale School of Public Health, New Haven, Connecticut; 5 Boston University Schools of Public Health and Medicine, Departments of Epidemiology, Biostatistics, Global Health and Medicine, Boston, Massachusetts; 6 Center for Health Policy, Stanford University, Stanford, California

## Abstract

**Importance:**

Despite significant progress made toward tuberculosis (TB) elimination, racial and ethnic disparities persist in TB incidence and case-fatality rates in the US.

**Objective:**

To estimate the health outcomes and economic cost of TB disparities among US-born persons from 2023 to 2035.

**Design, Setting, and Participants:**

Generalized additive regression models projecting trends in TB incidence and case-fatality rates from 2023 to 2035 were fit based on national TB surveillance data for 2010 to 2019 in the 50 US states and the District of Columbia among US-born persons. This baseline scenario was compared with alternative scenarios in which racial and ethnic disparities in age- and sex-adjusted incidence and case-fatality rates were eliminated by setting rates for each race and ethnicity to goal values. Additional scenarios were created examining the potential outcomes of delayed reduction of racial and ethnic disparities. The potential benefits of eliminating disparities from differences between baseline and alternative scenario outcomes were quantified. Data were analyzed from January 2010 to December 2019.

**Exposures:**

Non-Hispanic American Indian or Alaska Native, non-Hispanic Asian, non-Hispanic Black, Hispanic, non-Hispanic Native Hawaiian or Other Pacific Islander, or non-Hispanic White race and ethnicity.

**Main outcomes and measures:**

TB cases and deaths averted, quality-adjusted life years gained, and associated costs from a societal perspective.

**Results:**

The study included 31 811 persons with reported TB from 2010 to 2019 (mean [SD] age, 47 [24] years; 20 504 [64%] male; 1179 [4%] American Indian or Alaska Native persons; 1332 [4%] Asian persons; 12 152 [38%] Black persons; 6595 [21%] Hispanic persons; 299 [1%] Native Hawaiian or Other Pacific Islander persons; and 10 254 [32%] White persons). There were 3722 persons with a reported TB death. Persistent racial and ethnic disparities were associated with an estimated 11 901 of 26 203 TB cases among US-born persons (45%; 95% uncertainty interval [UI], 44%-47%), 1421 of 3264 TB deaths among US-born persons (44%; 95% UI, 39%-48%), and an economic cost of $914 (95% UI, $675-$1147) million from 2023 to 2035. Delayed goal attainment reduced the estimated avertable TB outcomes by 505 (95% UI, 495-518) TB cases, 55 (95% UI, 51-59) TB deaths, and $32 (95% UI, $24-$40) million in societal costs annually.

**Conclusions and relevance:**

In this modeling study of racial and ethnic disparities of TB, these disparities were associated with substantial future health and economic outcomes of TB among US-born persons without interventions beyond current efforts. Actions to eliminate disparities may reduce the excess TB burden among these persons and may contribute to accelerating TB elimination within the US.

## Key Points

**Question:**

What are the estimated future health and economic costs of tuberculosis (TB) if current trends in disparities by race and ethnicity in the US-born population persist?

**Findings:**

In this decision analytical model incorporating 31 811 individuals with TB, which assumes a continuation of current trends in racial and ethnic disparities, these disparities are associated with an estimated 11 901 of 26 203 TB cases (45%), 1421 of 3264 TB deaths (44%), and an economic cost of $914 million over the 2023 to 2035 period.

**Meaning:**

These findings suggest that without additional interventions, racial and ethnic disparities in TB are estimated to be associated with substantial future health and economic outcomes among US-born persons.

## Teaser

This decision analytical model evaluates the estimated health and economic outcomes of tuberculosis disparities among US-born persons from 2023 to 2035.

## Introduction

Significant progress has been made toward tuberculosis (TB) elimination in the US, resulting in one of the lowest TB incidence rates in the world.^1^ From 2010 to 2019, US TB incidence fell 25% to a rate of 2.7 cases per 100 000 persons, with a larger 44% decline among US-born persons, reaching a rate of 0.9 per 100 000 persons.^2^ TB disease arises from the progression of previously acquired *Mycobacterium tuberculosis (Mtb)* infection or a recent transmission event, with an estimated 13% of TB among US-born persons attributable to recent transmission from 2018 to 2019.^2,3^ TB case-fatality rates remained stable from 2010 to 2019.^2^ Despite these achievements in low TB incidence, transmission, and case-fatality rates, substantial racial and ethnic disparities persist in both TB incidence and case-fatality among US-born persons.^3,4,5^

Health disparities are plausibly avoidable systematic health differences that are closely linked with social, demographic, economic, and/or environmental disadvantage; these disadvantages may be historic or ongoing.^6,7^ Racial and ethnic disparities in TB could result from a number of mechanisms, including increased risk of exposure to the bacterium that causes TB (*Mtb*), elevated prevalence of risk factors for progression of latent tuberculosis infection (LTBI) to TB disease, reduced access to prevention services, delayed linkages to medical care, and a lower quality of care.^4,8,9^ A previous study analyzing US TB incidence from 2011 to 2021 estimated that US-born non-Hispanic Asian, non-Hispanic Black, Hispanic, and non-Hispanic American Indian or Alaska Native persons experienced substantially higher incidence rates than US-born non-Hispanic White persons.^3^ This study also found that the percentage of cases attributable to recent transmission varied by race and ethnicity, from 11% among non-Hispanic White persons to 31% among non-Hispanic American Indian or Alaska Native persons.^3^ During the same period, many of these minoritized persons also experienced elevated risks of death from TB compared with non-Hispanic White persons.^5^

The US Centers for Disease Control and Prevention (CDC) Division of Tuberculosis Elimination endeavors to reduce TB morbidity in the US with a particular focus on reducing disparities among disproportionately affected populations, including non–US-born and minoritized persons.^10^ The CDC’s National TB Performance targets are measurable, aspirational, and intended to be used by TB programs to assess their performance in achieving national program objectives. Targets have been set to reduce racial and ethnic disparities, including reduction in TB disease incidence among all US-born persons to 0.4 cases per 100 000 by 2025.^11^

Here, we estimated the potential health and societal costs of racial and ethnic disparities in TB among US-born persons in the absence of interventions beyond current TB elimination efforts. To do so, we projected trends in TB cases and deaths from 2023 to 2035 and compared this baseline trend with alternative scenarios in which incidence rates and case-fatality rates for each US-born race and ethnicity are reduced to target rates to eliminate disparities in these outcomes. We limited our analyses to US-born persons, as this aligns with the existing performance target for non-Hispanic Black persons and could be relevant for other races and ethnicities. We explored the potential health outcomes and societal costs that could be averted by eliminating these disparities.

## Methods

### Tuberculosis and Demographic Data

We extracted data from the National Tuberculosis Surveillance System (NTSS) for all US-born persons diagnosed with TB from 2010 to 2019 in the 50 US states and District of Columbia.^12^ Because of the impact of the COVID-19 pandemic from 2020 to 2022, we excluded that period. The CDC defines US-born persons as those who are entitled to US citizenship at birth.^12^ We retained variables describing individual race, ethnicity, age, year the case was counted, and whether the person survived the TB episode. We considered an individual as having died with TB if they were diagnosed with TB postmortem or if they were recorded as having died while receiving TB treatment, hereafter referred to as TB death. We categorized persons into 6 racial and ethnic groups: non-Hispanic American Indian or Alaska Native, non-Hispanic Asian, non-Hispanic Black, Hispanic, non-Hispanic Native Hawaiian or Other Pacific Islander, and non-Hispanic White, following US Census Bureau conventions (hereafter referred to as American Indian or Alaska Native, Asian, Black, Hispanic, Native Hawaiian or Other Pacific Islander, and White, respectively).^13^ We extracted data from the American Community Survey 2010 to 2019 5-year public-use microdata and calculated population estimates by race and ethnicity, sex, age in years, and calendar year.^14^ In accordance with 45 CFR §46, this study was exempt from the need for institutional board review and informed consent. This study is reported as per the Consolidated Health Economic Evaluation Reporting Standards (CHEERS) reporting guideline.^15^

### Baseline Incidence and Case-Fatality Rate Projections

We used generalized additive regression models fit to data from 2010 to 2019 to estimate baseline trends in TB incidence and case-fatality (probability of dying with TB) rates from 2023 to 2035.^16^ We used thin-plate regression splines to allow smooth differences in rates as a function of age and fit log-linear time trends for each race and ethnicity with fixed effects for sex.^17^ We omitted data for 2020 to 2022 when fitting these baseline trends, as these years were affected by the COVID-19 pandemic and may not be representative of the general time trends.^18^ We used the fitted regression models to project incidence and case-fatality rates from 2023 to 2035 and combined these with population projections to estimate total numbers of TB cases and deaths for each race and ethnicity. The results of these baseline projections are presented as total cases and deaths in this analysis. Given the observed variation and small sample size of TB cases among the non-Hispanic Native Hawaiian or Other Pacific Islander population with TB from 2010 to 2019, we fit supplemental models for this race and ethnicity using data over 2000 to 2019 (eAppendix 1, eFigure 1, and eFigure 2 in Supplement 1).

### Racial and Ethnic Disparity Elimination Scenarios

The CDC has a national TB program performance target for 2025 to reduce total US-born TB incidence to 0.4 cases per 100 000.^11^ We used this target as the goal rate for the incidence disparity analysis. As no target exists for TB case-fatality rates in the US, we used the lowest estimated age and sex adjusted case-fatality rate in 2023 as the target rate for our analysis.

To estimate the number of TB cases associated with racial and ethnic disparities in TB incidence, we compared incidence estimated for the baseline scenario with an alternative scenario in which each race and ethnicity reached the target incidence rate of 0.4 cases per 100 000 in 2023 (incidence disparities eliminated scenario). This change was implemented by assuming a proportional reduction in incidence rates within each year of age and sex stratum, occurring in 2023, such that average age- and sex-adjusted incidence for each race and ethnicity matched the target rate. After 2023, incidence rates for each race and ethnicity were assumed to follow the same annual percentage reduction as in the baseline scenario. We recalculated TB cases and deaths from 2023 to 2035 for this incidence disparities eliminated scenario. The difference between these estimates and those calculated for the baseline represent the estimated number of TB cases and deaths associated with racial and ethnic disparities in TB incidence from 2023 to 2035.

To estimate the outcomes associated with racial and ethnic disparities in TB case-fatality rates, we created a case-fatality disparities eliminated scenario, in which the age- and sex-adjusted case-fatality rate for each population was reduced to match the target case-fatality rate in 2023 (lowest estimated age- and sex-adjusted case-fatality rate in 2023; reported in results). The difference between TB deaths estimated under this scenario and those calculated for the baseline represent the estimated number of TB deaths associated with racial and ethnic disparities in TB case-fatality from 2023 to 2035. Finally, to estimate the combined outcomes associated with racial and ethnic disparities in TB incidence and case-fatality rates, we created a third scenario (both disparities eliminated) combining the incidence and case-fatality rate reductions of the 2 other alternative scenarios and calculated the difference in TB cases and deaths under this scenario compared with the baseline.

### Examining the Outcomes of Delayed Target Attainment

We created additional alternative scenarios by starting the disparity reduction period in 2023 then varying the year in which racial and ethnic disparities in incidence and case-fatality rates would be eliminated. We operationalized these scenarios as log-linear declines parameterized to achieve target incidence and case-fatality rates across a range of years (2023, 2024, …, 2035). In these scenarios, we assumed the annual percentage change in each rate would return to that of the baseline after the target rate was achieved. We compared TB cases and deaths from these scenarios with those projected for the other scenarios and used these results to describe the benefits that could be achieved with delayed racial and ethnic disparity elimination.

### Summary Health and Economic Outcome

We calculated additional outcomes to describe the health and economic losses associated with racial and ethnic disparities over the study period. First, we calculated quality-adjusted life years (QALYs) as a summary measure of changes in health. These were calculated as the sum of QALYs lost due to deaths with TB and nonfatal health reductions during TB disease and treatment. We also present average QALYs gained per TB case averted for each race and ethnicity.^19^ Second, we calculated the economic cost of TB incidence and case-fatality reductions under a societal perspective, considering future changes in TB health services costs, non-TB health care spending, and productivity, adjusted for future non–health care spending. eAppendix 2, eTable 1, and eTable 2 in Supplement 1 present further detail on the economic analysis methods.

### Statistical Analysis

All age- and sex-adjusted incidence and case-fatality rates were standardized to the US-born population and TB case distributions, respectively. We estimated 95% uncertainty intervals (UI) for each study outcome using a Monte Carlo approach, simulating 1000 parameter sets from the fitted regression coefficients, and reporting UIs as the 2.5th and 97.5th percentiles.^20^ All analyses were conducted in R version 4.2.1 (R Project for Statistical Computing) using the mgcv version 1.8.40 and MASS version 7.3.60 packages.^21,22,23^ Data were analyzed from January 2010 to December 2019.

## Results

### Baseline Health Outcomes of TB

The study included 31 811 persons with a reported TB case in 2010 to 2019 (mean [SD] age, 47 [24] years; 20 504 [64%] male), with 1179 (4%) American Indian or Alaska Native persons; 1332 (4%) Asian persons; 12 152 (38%) Black persons; 6595 (21%) Hispanic persons; 299 (1%) Native Hawaiian or Other Pacific Islander persons; and 10 254 (32%) White persons. A total of 3722 persons had a reported TB death (mean [SD] age, 67 [17] years; 2536 [68%] male), with 155 (4%) American Indian or Alaska Native persons; 58 (2%) Asian persons; 1559 (42%) Black persons; 490 (13%) Hispanic persons; 17 (<1%) Native Hawaiian or Other Pacific Islander persons; and 1447 (39%) White persons.

**Figure 1.**
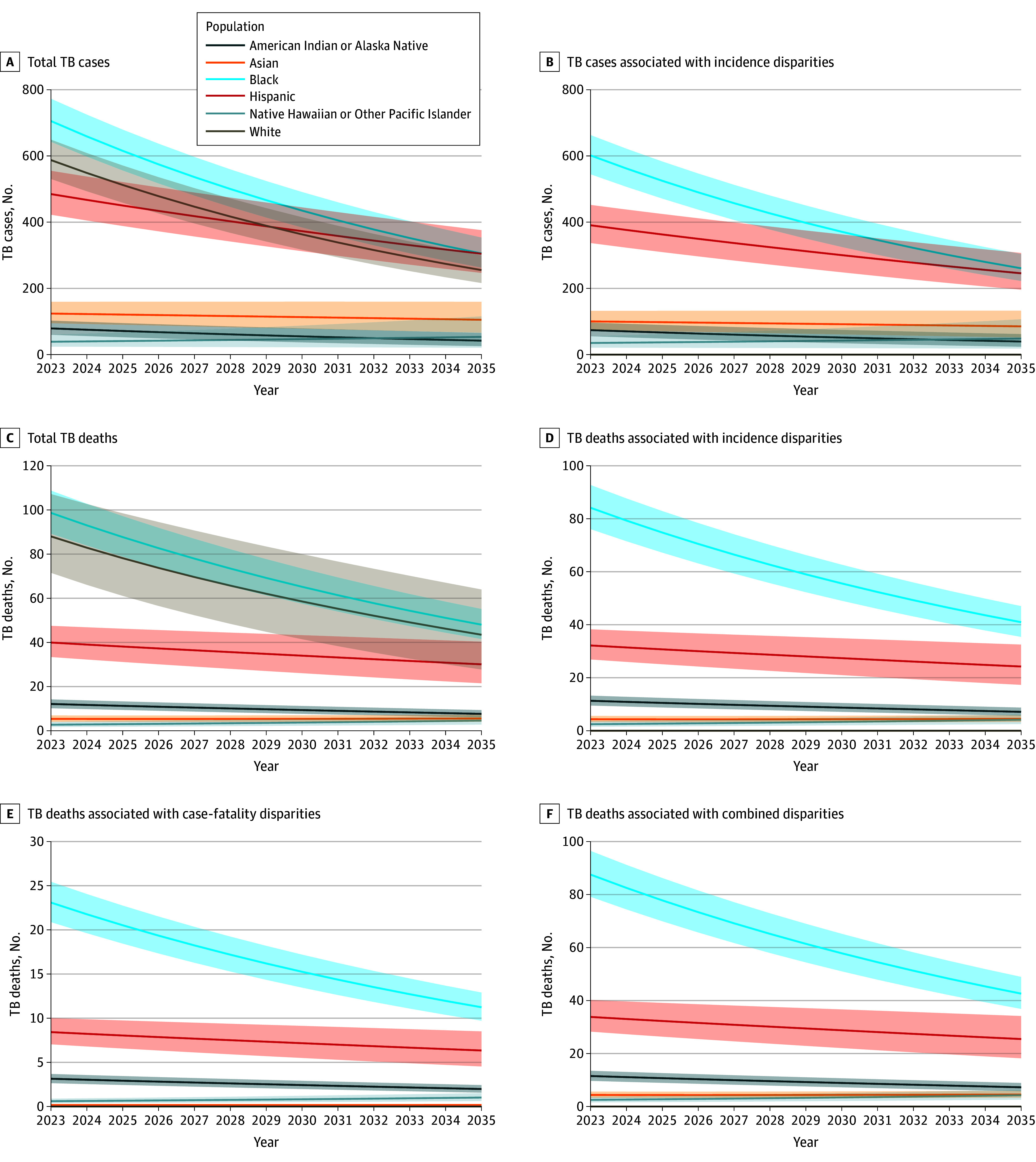
Race and Ethnicity Stratified Trends in Tuberculosis (TB) Cases and Deaths Among US-Born Persons From 2023 to 2035: Total and Associated With Racial and Ethnic Disparities Panels labeled “Total TB cases” and “Total TB deaths” show the baseline model trends. Shaded areas indicate 95% uncertainty intervals.

Figure 1 presents estimated trends in annual TB cases and deaths under the baseline scenario. In this scenario, we estimate there will be 26 203 (95% UI, 24 931-27 564) TB cases and 3264 (95% UI, 3051-3513) TB deaths among US-born persons between 2023 and 2035. Over this period, estimated incidence rates among US-born persons fell by 49% (95% UI, 46%-52%), from 0.72 (95% UI, 0.70-0.75) to 0.37 (95% UI, 0.34-0.41) per 100 000 persons; estimated average case-fatality rates increased by 7% (95% UI, 3%-12%), from 12.21 (95% UI, 11.50-12.98) to 13.06 (95% UI, 11.94-14.48) per 100 cases, following estimated changes in the age distribution of TB cases (eFigure 3 in Supplement 1).

eFigure 4 in Supplement 1 shows the estimated age- and sex-adjusted incidence and case-fatality rates for each race and ethnicity. The lowest estimated adjusted case-fatality rate was among White persons, which remained stable at approximately 10 deaths per 100 cases between 2023 and 2035. This case-fatality rate (0.10) was used as the target, and we did not analyze a change in case-fatality rate among White persons.

eTable 3 in Supplement 1 presents the distribution of cumulative QALYs lost to TB across each race and ethnicity. From the baseline projections we estimated an average 2.0 (95% UI, 1.8-2.1) QALYs lost per TB case, totaling 51 762 (95% UI, 48 429-55 212) QALYs from 2023 to 2035. Further baseline results can be found in the eResults in Supplement 1. Black and non-Hispanic American Indian or Alaska Native persons had the highest QALYs lost per case (2.4 and 2.3, respectively), and Asian persons had the lowest (1.3).

### Health Outcomes of Racial and Ethnic Disparities in TB

**Figure 2.**
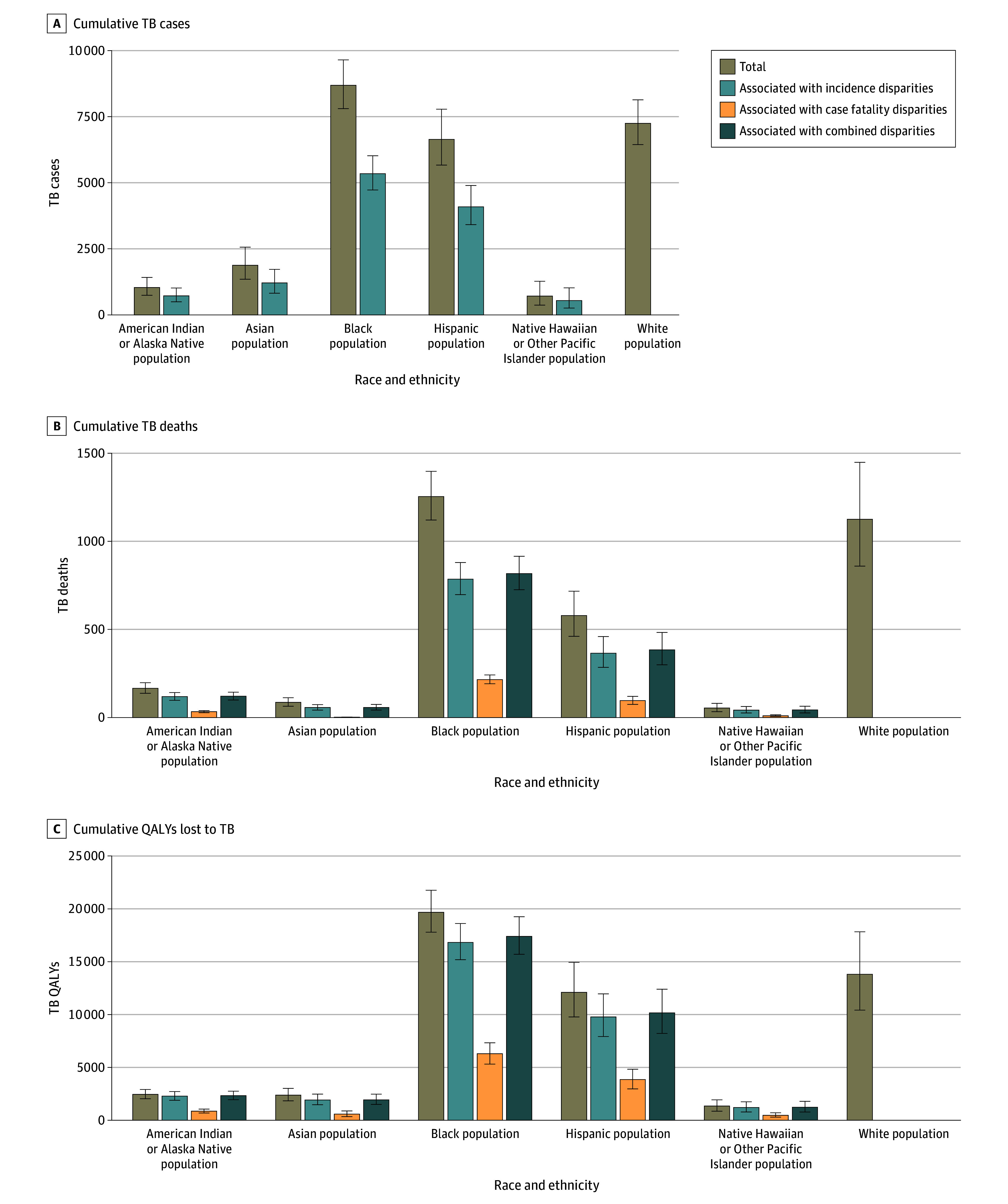
Race and Ethnicity Stratified Cumulative Tuberculosis (TB) Cases, TB Deaths, and Quality-Adjusted Life-Years (QALYs) Lost to TB Among US-Born Persons From 2023 to 2035: Total and Associated With Racial and Ethnic Disparities Bars labeled “Total” show the cumulative baseline model outcomes. Error bars indicate 95% uncertainty intervals.

Figure 2 depicts the estimated race and ethnicity stratified cumulative TB cases and deaths associated with racial and ethnic disparities in TB incidence and case-fatality from 2023 to 2035. Black persons were estimated to experience the greatest number of TB cases associated with racial and ethnic disparities (5342; 95% UI, 4724-6022 cases). Black persons were also projected to experience the highest number of deaths associated with racial and ethnic disparities with 785 (95% UI, 716-858) deaths associated with incidence disparities and 215 (95% UI, 197-235) deaths associated with case-fatality disparities. In the both disparities eliminated scenario, an estimated 1421 (95% UI, 1307-1543) deaths were associated with racial and ethnic disparities.

eTable 3 in Supplement 1 compares race and ethnicity–stratified TB cases, deaths, and QALYs under the baseline and associated with racial and ethnic disparities. Between 2023 and 2035, we estimated 45% (95% UI, 44%-47%) of TB cases among US-born persons would be associated with racial and ethnic disparities in TB incidence rates. Non-Hispanic Native Hawaiian or Other Pacific Islander persons had the highest proportion (75%; 95% UI, 68%-81%) of disparity-associated cases. Among all race and ethnicities, 42% (95% UI, 37%-46%) and 11% (95% UI, 10%-12%) of estimated TB deaths among US-born persons were associated with disparities in TB incidence and case-fatality rates, respectively. Proportions were highest among non-Hispanic Native Hawaiian or Other Pacific Islander persons with 78% (95% UI, 77%-78%) of estimated deaths associated with incidence disparities and among non-Hispanic American Indian or Alaska Native persons with 20% (95% UI, 20%-20%) associated with case-fatality disparities.

eFigure 5 in Supplement 1 shows the age distribution of cumulative TB cases and deaths averted under the both disparities eliminated scenario. Asian persons had the largest proportion of cases in those aged 25 to 34 years (26%; 95% UI, 24%-28%). Non-Hispanic American Indian or Alaska Native and Black persons had the largest proportion of TB cases among those aged 60 to 100 years (44%; 95% UI, 41%-47%; and 39%; 95% UI, 38%-40%, respectively). The majority of TB deaths were among persons at least 60 years of age in each race and ethnicity, ranging from 58% (95% UI, 55%-60%) among Asian persons to 77% (95% UI, 75%-78%) of deaths among Non-Hispanic American Indian or Alaska Native persons.

### Societal Cost of Racial and Ethnic Disparities in TB

The Table shows the estimated societal costs due to disparities in TB among US-born persons from 2023 to 2035. Under the baseline scenario, we estimated societal costs due to TB disease would be US $1.397 (95% UI, $1.092-$1.691) billion. The estimated percentage of these societal costs associated with incidence disparities was 64% (95% UI, 47%-87%) at $892 (95% UI, $679-$1132) million; case-fatality disparities was 47% (95% UI, 36%-62%) at $650 (95% UI, $514-$785) million USD; and combined disparities was 66% (95% UI, 45%-89%) at $914 (95% UI, $675-$1147) million.

**Table.** Cumulative Economic Burden (in Billions) Associated With the Baseline and Excess Tuberculosis (TB) Morbidity and Mortality Due to Racial and Ethnic Disparities in TB Incidence and Case-Fatality Among US-Born Persons, 2023 to 2035

Scenario	TB health services costs		Non-TB health services costs		Productivity costs		Societal costs	
	Total, US $ (95% UI)	% of Baseline (95% UI)	Total, US $ (95% UI)	% of Baseline (95% UI)	Total, US $ (95% UI)	% of Baseline (95% UI)	Total, US $ (95% UI)	% of Baseline (95% UI)
Baseline	0.386 (0.294 to 0.484)	100	−0.056 (−0.072 to −0.042)	100	1.067 (0.776 to 1.341)	100	1.397 (1.092 to 1.691)	100
Associated with								
Incidence disparity	0.235 (0.167 to 0.309)	62 (40 to 89)	−0.031 (−0.041 to −0.021)	157 (135 to 183)	0.688 (0.492 to 0.925)	64 (43 to 95)	0.892 (0.679 to 1.132)	64 (47 to 87)
Case-fatality disparity	0	NA	−0.008 (−0.011 to −0.006)	115 (109 to 123)	0.273 (0.181 to 0.373)	26 (16 to 39)	0.650 (0.514 to 0.785)	47 (36 to 62)
Incidence and case-fatality disparity	0.235 (0.167 to 0.309)	62 (40 to 89)	−0.033 (−0.043 to −0.023)	160 (139 to 189)	0.712 (0.489 to 0.937)	68 (42 to 99)	0.914 (0.675 to 1.147)	66 (45 to 89)

Abbreviation: NA, not applicable.

### Consequences of Delayed Target Attainment

**Figure 3.**
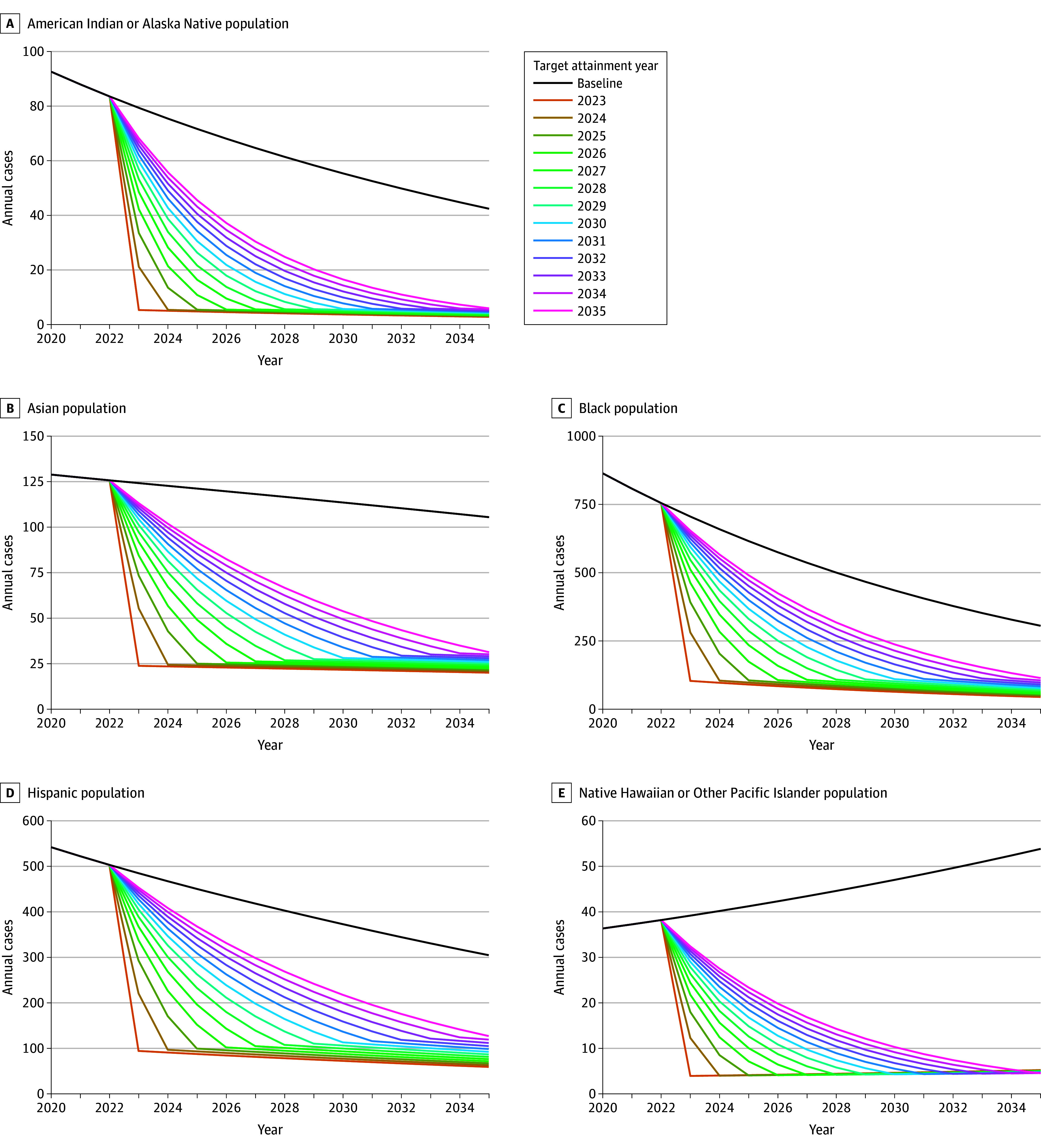
Annual Tuberculosis Cases for Different Disparity Goal Attainment Years Under the Both Disparities Removed Scenario Compared With the Baseline Among US-Born Persons From 2023 to 2035 for Each Race and Ethnicity

**Figure 4.**
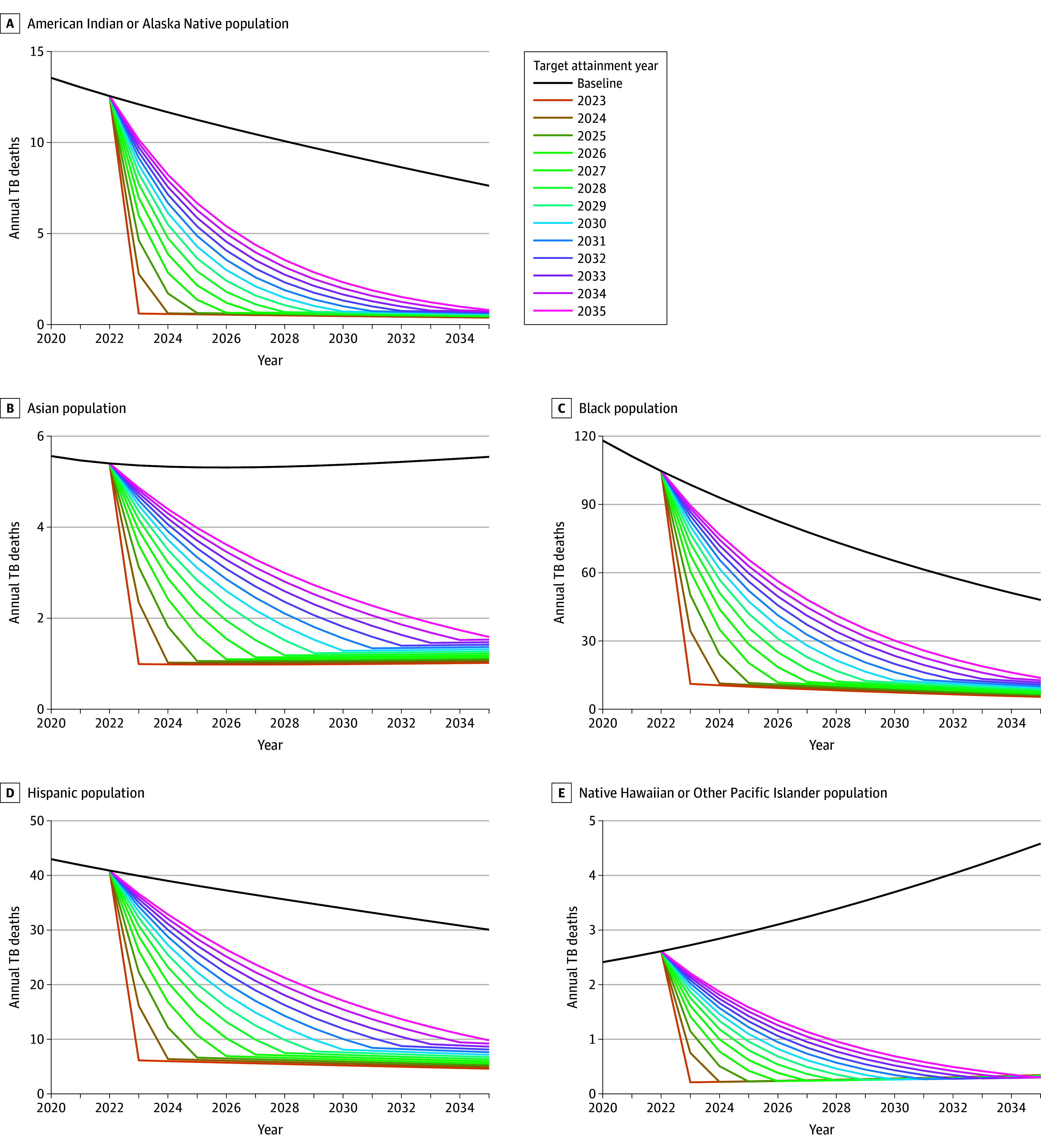
Annual Tuberculosis (TB) Deaths for Different Disparity Goal Attainment Years Under the Both Disparities Removed Scenario Compared With the Baseline Among US-Born Persons From 2023 to 2035 for Each Race and Ethnicity

Figures 3 and 4 show trends in TB cases and deaths by race and ethnicity, respectively, for different target attainment years, under the both disparities eliminated scenario compared with baseline projections. We estimated a reduction of 505 (95% UI, 495-518) TB cases and 55 (95% UI, 51-59) TB deaths averted with each year of delayed target attainment across all races and ethnicities under the both disparities eliminated scenario. We estimated cumulative QALYs lost to TB would be reduced by 33 051 (95% UI, 30 181-36 277) QALYs if targets were met in 2023 and by 16 053 (95% UI, 14 534-17 796) cumulative QALYs if targets were only met in 2035.

Delayed target attainment reduced the estimated averted societal cost of disparity reductions at a rate of $32 (95% UI, $24-$40) million annually, as well as 1307 QALYs. The societal cost averted by removing disparities by 2035 was $412 (95% UI, $310-$519) million, which is 42% (95% UI, 29%-60%) of the estimated reduction of removing these TB disparities by 2023.

## Discussion

We estimated the health outcomes and societal costs of TB among US-born persons associated with racial and ethnic disparities from 2023 to 2035 in this decision analytical model. If current trends in TB incidence and case-fatality rates continue, nearly half of all projected TB cases and deaths among all US-born persons and approximately two-thirds of TB cases and deaths among people in minoritized populations will be associated with racial and ethnic disparities. Cumulatively, these cases and deaths were estimated to be responsible for the majority of QALYs lost to TB and societal costs of TB among US-born persons from 2023 to 2035. These estimated TB health outcomes associated with racial and ethnic disparities incur a sizable societal cost, with the combined cost of incidence and case-fatality disparities accounting for nearly $1 billion, or 66% of the total estimated cost of TB disease during the same period ($1.4 billion).

Among US-born persons, Black persons had the highest estimated total QALYs lost to TB associated with racial and ethnic disparities, suggesting that efforts focused on this population may have the largest impact toward the reduction of TB burden while furthering equity goals. Non-Hispanic Native Hawaiian or Other Pacific Islander persons had the largest estimated proportion of racial and ethnic disparity–associated QALYs lost. Despite having a smaller cumulative outcome, it is essential to identify interventions focused on this population, as they experience the greatest disproportionate impact of racial and ethnic disparities of TB.

US-born populations differed in their age distribution of TB cases associated with racial and ethnic disparities. These differences, along with those in age- and sex-adjusted case-fatality rates, resulted in the variance of estimated average QALYs lost per TB case across races and ethnicities. Among both Black and non-Hispanic American Indian or Alaska Native persons, approximately 40% of cases were estimated to occur among persons 60 years or older, suggesting that many of these cases may result from progression of latent TB infection.^24^ These populations were also estimated to have the highest adjusted case-fatality rates, resulting in the highest QALYs lost per case. In contrast, among Asian persons, 26% of TB cases were estimated to occur in those aged 25 to 34 years, which was a key factor in the Asian population’s 1.3 estimated QALYs lost per case. Most TB deaths were estimated to be among persons at least 60 years old across all races and ethnicities.

We further examined what fraction of TB burden could be averted under different years of target attainment. Steady progress beginning in 2023 achieving parity in TB incidence and case-fatality in 2035 resulted in a total 16 053 QALYs gained compared with the baseline scenario. These QALYs account for 49% of the total QALYs lost to TB due to racial and ethnic disparities from 2023 to 2035. A total of 1307 QALYs and $32 million USD in societal costs were associated with each year of inaction.

Our baseline incidence projections showed slow TB incidence declines among US-born persons, consistent with previous mathematical modeling studies.^25,26,27^ These projections include decreasing magnitude of incidence disparities among all persons and stagnant incidence rates projected for Native Hawaiian or Other Pacific Islander persons. Yet, despite these reductions, age- and sex-adjusted incidence rates among all US-born persons, except among White persons, will not reach the current national target of 0.4 case per 100 000 persons by 2035 under the baseline scenario.^28^ These baseline estimates were based on data before the COVID-19 pandemic. Recent TB surveillance data suggest that pandemic-related changes may have had a differential impact on TB incidence rates among US-born persons by race and ethnicity. During 2020 to 2023, US-born American Indian or Alaska Native, Asian, and Native Hawaiian or Other Pacific Islander persons experienced elevated reported TB incidence as compared with 2019. In contrast, US-born Black persons had reduced rates.^29^ The durability of these observed changes is uncertain; however, any long-term impact of the COVID-19 pandemic or any future pandemic could alter the impact on racial and ethnic TB disparities.

### Limitations

This study has limitations. The estimated baseline TB case and death projections and cost calculations are based on inputs that are uncertain. We attempted to reflect the consequences of uncertainty in these inputs by allowing uncertainty to propagate throughout each step of the analytical approach. Additionally, our analysis only accounted for the outcomes of disparities through 2035. While the bulk of the benefit of reducing disparities would be in the years immediately following parity, achieving equity would also produce sustained health benefits. Furthermore, our analysis relies on reported case and death data, which only capture those who have had contact with the health system during their TB episode. Some minoritized persons have higher barriers to medical care, which may result in underdiagnosis of disease.^30^ These potential omissions may result in a downward bias in our estimation. Finally, we assume all estimated changes in mortality are associated with the modeled changes in incidence and case-fatality rates, even though a fraction of TB deaths will be due to other causes.^31^

Our analysis does not consider the underlying mechanisms that produce the estimated disparities in TB incidence and case-fatality, but rather estimated the health and societal cost of racial and ethnic disparities in TB due to all causes. Understanding the estimated future outcomes of these disparities provides support for current and future interventions to reduce disparities. Additional research on the mechanisms underlying racial and ethnic disparities is urgently needed to inform policies that may result in their reduction and hasten progress toward both health equity and TB elimination goals.

If current trends continue, racial and ethnic disparities in TB will produce significant health outcomes and societal costs for minoritized persons in coming decades. Identification and implementation of interventions focused on the drivers of disparities for all racial and ethnic groups is crucially needed to achieve equity goals. These efforts will need to address TB disease arising from LTBI reactivation as well as *Mtb* transmission in the US. Reducing racial and ethnic disparities in TB among US-born persons could attenuate these health and economic losses and accelerate progress toward TB elimination in the US.

## Conclusions

To achieve health equity in TB in the US, racial and ethnic disparities in TB incidence and case-fatality rates must be addressed. Without more dedicated efforts to eliminate these disparities, they could be associated with major health and economic losses in coming years among US-born persons who already face higher levels of social and economic marginalization. Actions to close racial and ethnic disparities may reduce the excess TB burden among these persons and contribute to accelerating TB elimination within the US.

## Notes

Supplement 1. **eAppendix 1. **Supplemental Native Hawaiian or Other Pacific Islander Model Fitting **eFigure 1. **Age- and Sex-Adjusted TB Incidence Rates During 2023 to 2035 Among US-born Native Hawaiian or Other Pacific Islander Persons Produced Using Extended Data **eFigure 2. **Age- and Sex-Adjusted TB Case-Fatality Rates During 2023 to 2035 Among US-Born Native Hawaiian or Other Pacific Islander Persons Produced Using Extended Data **eAppendix 2.** Economic Analysis Methods **eTable 1. **Inputs for QALY Calculation **eTable 2. **Inputs for Productivity Costs Associated With TB Disease **eFigure 3.** Baseline Age Distribution of TB Cases and Deaths in 2023 and 2035 **eFigure 4. **Baseline Projections of Age- and Sex-Adjusted Rates Among US-Born Persons Between 2023 and 2035 **eTable 3. **Estimated Baseline and Excess TB Morbidity and Mortality Associated With Racial and Ethnic Disparities in TB Incidence and Case-Fatality Among US-Born Persons, 2023 to 2035 **eFigure 5. **Age Distribution of Cumulative TB Cases and Deaths Averted Among US-Born Persons During 2023 to 2035 Under the Both Disparities Removed Scenario for Each Race and Ethnicity
eResults.

Supplement 2.
Data Sharing Statement
