# Supplementary material for: Estimated Health and Economic Outcomes of Racial and Ethnic Tuberculosis Disparities in US-Born Persons
Source: JAMA Netw Open. 2024 Sep 10;7(9):e2431988. doi: 10.1001/jamanetworkopen.2024.31988 (PMC11388029; doi:10.1001/jamanetworkopen.2024.31988)
Supplement: Supplement 1. — eAppendix 1. Supplemental Native Hawaiian or Other Pacific Islander Model Fitting eFigure 1. Age- and Sex-Adjusted TB Incidence Rates During 2023 to 2035 Among US-born Native Hawaiian or Other Pacific Islander Persons Produced Using Extended Data eFigure 2. Age- and Sex-Adjusted TB Case-Fatality Rates During 2023 to 2035 Among US-Born Native Hawaiian or Other Pacific Islander Persons Produced Using Extended Data eAppendix 2. Economic Analysis Methods eTable 1. Inputs for QALY Calculation eTable 2. Inputs for Productivity Costs Associated With TB Disease eFigure 3. Baseline Age Distribution of TB Cases and Deaths in 2023 and 2035 eFigure 4. Baseline Projections of Age- and Sex-Adjusted Rates Among US-Born Persons Between 2023 and 2035 eTable 3. Estimated Baseline and Excess TB Morbidity and Mortality Associated With Racial and Ethnic Disparities in TB Incidence and Case-Fatality Among US-Born Persons, 2023 to 2035 eFigure 5. Age Distribution of Cumulative TB Cases and Deaths Averted Among US-Born Persons During 2023 to 2035 Under the Both Disparities Removed Scenario for Each Race and Ethnicity eResults. [file jamanetwopen-e2431988-s001.pdf]

Supplemental Online Content

Swartwood NA, Li Y, Regan M, et al. Estimated health and economic impacts of racial and ethnic tuberculosis disparities. *JAMA Netw Open*. 2024;7(9):e2431988. doi:10.1001/jamanetworkopen.2024.31988

eAppendix 1. Supplemental Native Hawaiian or Other Pacific Islander Model Fitting
eFigure 1. Age- and Sex-Adjusted TB Incidence Rates During 2023 to 2035 Among US-Born Native Hawaiian or Other Pacific Islander Persons Produced Using Extended Data
eFigure 2. Age- and Sex-Adjusted TB Case-Fatality Rates During 2023 to 2035 Among US-Born Native Hawaiian or Other Pacific Islander Persons Produced Using Extended Data
eAppendix 2. Economic Analysis Methods
eTable 1. Inputs for QALY Calculation
eTable 2. Inputs for Productivity Costs Associated With TB Disease
eFigure 3. Baseline Age Distribution of TB Cases and Deaths in 2023 and 2035
eFigure 4. Baseline Projections of Age- and Sex-Adjusted Rates Among US-Born Persons Between 2023 and 2035
eTable 3. Estimated Baseline and Excess TB Morbidity and Mortality Associated With Racial and Ethnic Disparities in TB Incidence and Case-Fatality Among US-Born Persons, 2023 to 2035
eFigure 5. Age Distribution of Cumulative TB Cases and Deaths Averted Among US-Born Persons During 2023 to 2035 Under the Both Disparities Removed Scenario for Each Race and Ethnicity
eResults.

This supplemental material has been provided by the authors to give readers additional information about their work.

© 2024 Swarwood NA et al. *JAMA Network Open.*

eAppendix 1. Supplemental Native Hawaiian or Other Pacific Islander Model Fitting

The 2010–2019 trend in reported TB cases among Native Hawaiian or Other Pacific Islander persons had few observations and high noise compared to the other examined race-ethnicities. We refit the incidence and case-fatality projection models using National Tuberculosis Surveillance System data and American Community Survey population data for an extended time period from 2000–2019. We then compared the resultant projections to those from our initial models. Figure S1 shows the projected incidence rates from this model. The aIR in 2023 using this revised incidence model is 6.78 (4.43 – 17.94) compared with the goal rate of 0.4 cases per 100,000 population, which is 80% higher than that in our original model. This increase would correspond to an additional 568 TB cases in our baseline projections.

Figure S2 shows the projected case-fatality rates from the model fit to data from 2000–2019. The aCFR in 2023 using this revised model is 1.35 (0.97 – 1.83) compared with the goal rate of 10 deaths per 100 cases, which is 5% higher than that in our original model. This increase would correspond to an additional 3 TB deaths in our baseline projections.

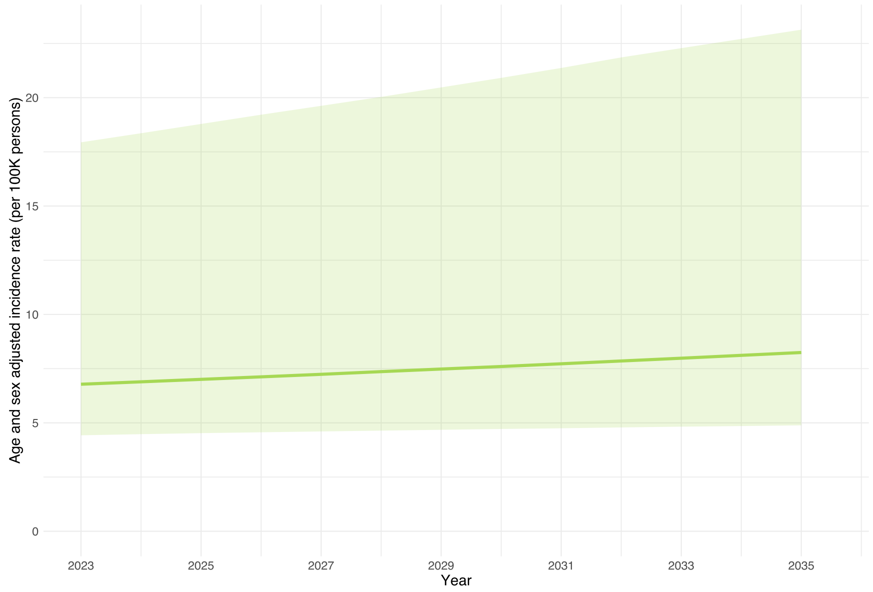

eFigure 1. Age- and Sex-Adjusted TB Incidence Rates During 2023 to 2035 Among US-Born Native Hawaiian or Other Pacific Islander Persons Produced Using Extended Data

© 2024 Swarwood NA et al. *JAMA Network Open.*

50

© 2024 Swarwood NA et al. *JAMA Network Open.*

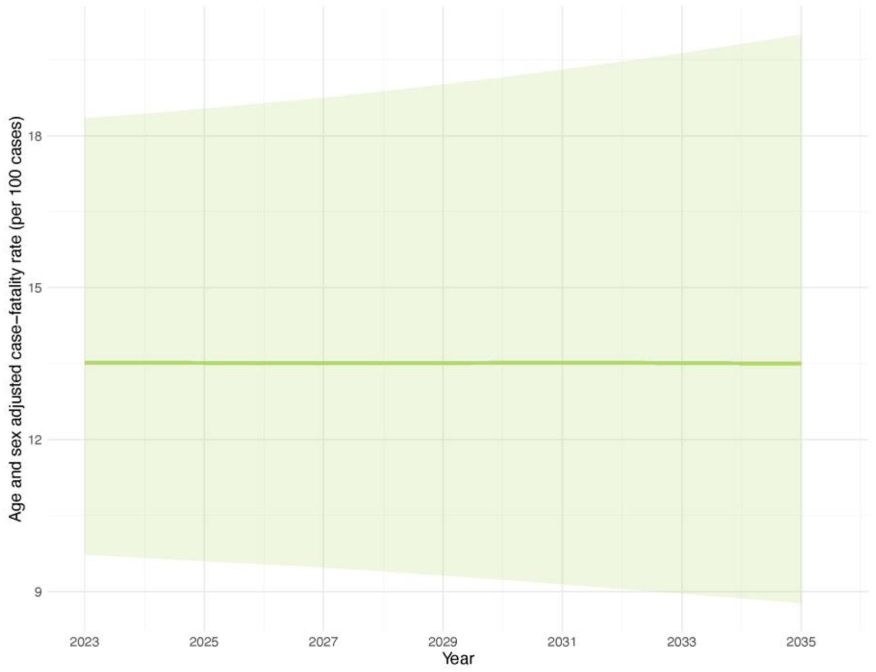

eFigure 2. Age- and Sex-Adjusted TB Case-Fatality Rates During 2023 to 2035 Among US-Born Native Hawaiian or Other Pacific Islander Persons Produced Using Extended Data

© 2024 Swarwood NA et al. *JAMA Network Open.*

eAppendix 2. Economic Analysis Methods

Parameter uncertainty

For each of the 1,000 model simulations, we sampled each cost and QALY calculation parameter from a probability distribution to account for uncertainty. For all of these parameters described below that had a published uncertainty range, we use these values to parameterize the distribution. For all other listed parameters, we assume an uncertainty range of $\pm$ 25%. These uncertainty ranges are listed alongside their point estimates below.

Health impacts

Quality adjusted life years (QALYs)

We first calculate the total QALYs lost to TB for each scenario. These are calculated as the sum of:

(i) *QALYs lost due to TB death*: these are calculated by taking the cumulative number of TB deaths in each age and multiplying that number by a life expectancy estimate for that age.[i] We then sum these totals across all ages.

(ii) *QALYs lost during TB treatment:* these are calculated as the product of time spent on TB treatment and the disutility of TB disease.

Values and sources for QALY inputs are shown in Table S1. Estimates of QALYs saved are calculated by subtracting the total QALYs lost to TB in a given scenario from the total QALYs lost to TB under a counterfactual scenario (e.g., the baseline). We assume that all observed QALY differences between scenarios are attributable to changes in TB incidence and TB case-fatality rates. QALY decrements do not include those due to long-term sequelae from TB disease. We also present average QALY gained per TB case averted, which is calculated as the sum of QALYs gained divided by the sum of TB cases averted for each race-ethnicity.

© 2024 Swarwood NA et al. *JAMA Network Open.*

eTable 1. Inputs for QALY Calculation

Input	Value (95% uncertainty interval)
Life expectancy at the time of TB death[i]	2.13–77.07 years
**Utility decrements**	
TB disease and treatment[ii]	0.24 (0.18–0.30)
**Other parameters**	
Duration of TB disease prior to TB treatment	3 months (2.25–3.75 months)
Duration of TB treatment	9 months* (6.75–15 months)

Notes: *A 6-month TB treatment regimen takes, on average, 9 months to complete due to interruptions in care.

Health services costs

Health services costs are calculated from incremental differences in health service provision estimated for each scenario and the baseline, cumulated over 2023–2035, and unit costs estimated for each of these services. Per-patient TB treatment costs were based on cost analyses conducted by the CDC.[7] Annual total healthcare expenditures by age were based on Medical Expenditure Panel Survey estimates.[23] Details on the calculation of each cost outcomes are shown below.

<u>Health service cost of TB disease treatment</u>

TB treatment costs are calculated as the number of individuals receiving TB treatment multiplied by a fixed unit cost of $23,060 (17,295–28,825).[iii] Of note, the model does not stratify cases by drug resistance and therefore assumes a single unit cost for TB treatment, representing the cost of treating drug susceptible TB case. The average cost of TB treatment includes the cost of diagnosing TB disease.

<u>Non-TB Future Healthcare costs</u>

For each disparity eliminated scenario, future healthcare costs are calculated for those persons surviving the TB episode who would not have under the baseline scenario. These persons incur costs that they would not have if they had not survived the episode; the sum of these costs are

© 2024 Swarwood NA et al. *JAMA Network Open.*

represented as a negative number in our analysis as they reduce the potential societal costs saved through increased TB survival. These costs are calculated by multiplying the deaths averted under a scenario by an estimated lifetime annual healthcare spending. These costs vary by single year of age and range from 2,141–18,586 (1,605–23,232).[iv]

Productivity costs

Economic productivity costs associated with receipt of TB treatment and TB disease morbidity and mortality are calculated from counts of health outcomes and health services estimated between each scenario and age-stratified estimates of economic productivity loss. Annual total non-healthcare expenditures were calculated from the U.S. Bureau of Labor Statistics' Consumer Expenditure Surveys.[24] Annual and lifetime productivity estimates were based on an analysis of U.S. market and non-market productivity.[25] Details on the calculation of these outcomes are shown below.

Productivity costs of TB disease

The productivity costs of TB disease are calculated as the sum of productivity losses from mortality and morbidity. Mortality costs for each scenario are calculated as loss of productivity given the age-specific life expectancy at TB death and the remaining age-specific annual productivity estimates, adjusted for future non-healthcare spending. Morbidity costs are calculated as the average time loss due to outpatient TB services and the average hospitalization cost, derived as the product of the probability of hospitalization, average duration of hospitalization, and age-specific productivity estimate. The total productivity costs of TB disease are the sum of both of these values. Values used in these calculations are shown in Table S2.

eTable 2. Inputs for Productivity Costs Associated With TB Disease

Input	Value (95% uncertainty interval)
Probability of hospitalization with TB[v]	0.49 (0.37–0.61)
Average duration of hospitalization with TB[vi]	24 days (18–30)
Time losses due to outpatient services[iv]	6.8 days (5.1–8.5)

© 2024 Swarwood NA et al. *JAMA Network Open.*

Annual productivity by single year of age[vii]	range: 0–99,843 (0–124,804)
Lifetime productivity by single year of age[v]	range: 0–4,432,248 (0–5,540,310)
Future non-healthcare spending[viii]	range: 4,621–1,538,447 (3,466–1,923,059)

Total societal cost estimates

The scenario-specific total societal cost is equal to the sum of health services costs and productivity costs over 2023–2035 for that scenario.

© 2024 Swarwood NA et al. *JAMA Network Open.*

A.

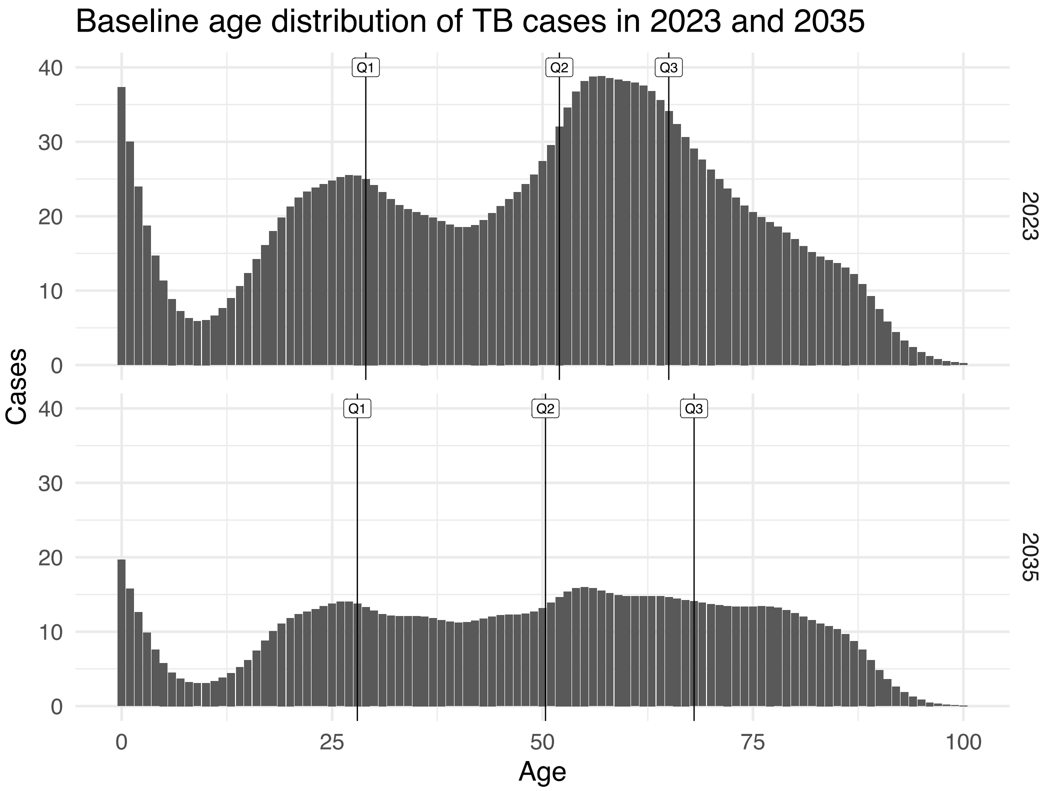

B.

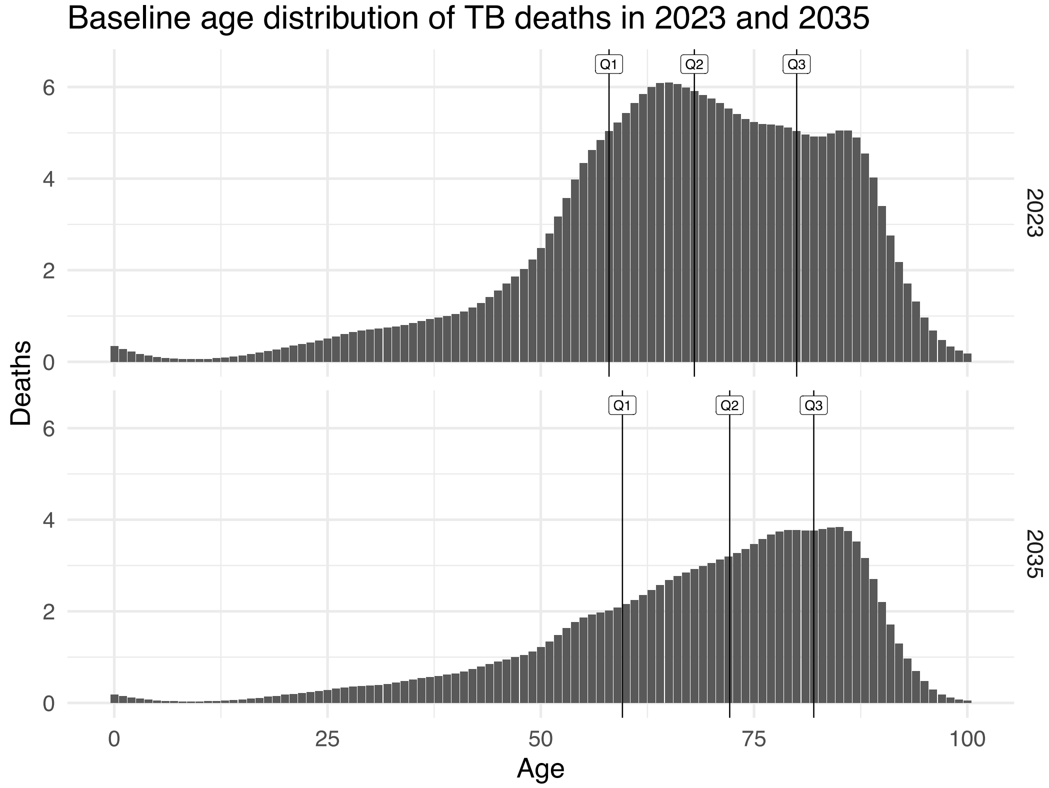

eFigure 3. Baseline Age Distribution of TB Cases and Deaths in 2023 and 2035 Panel A: Baseline age distribution of TB cases in 2023 and 2035; Panel B: Baseline age distribution of TB deaths in 2023 and 2035. Q1, Q2, and Q3 represent the first, second, and third quantile, respectively.

© 2024 Swarwood NA et al. *JAMA Network Open.*

A.

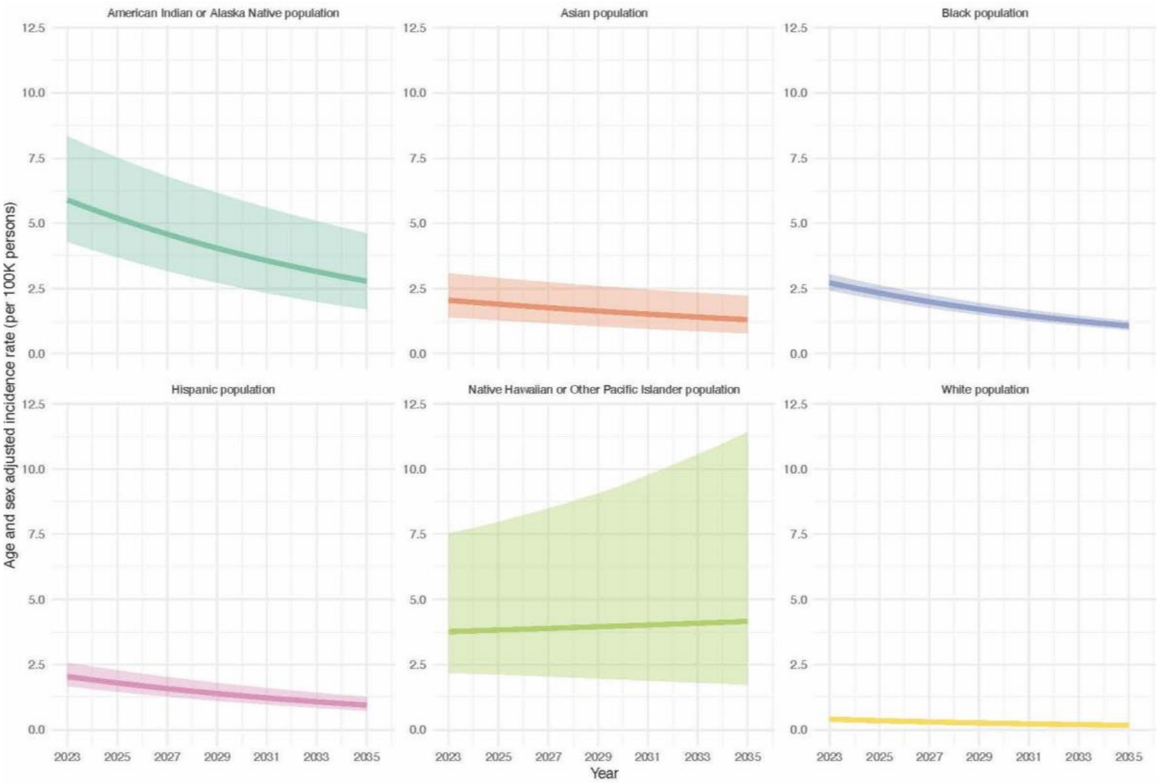

B.

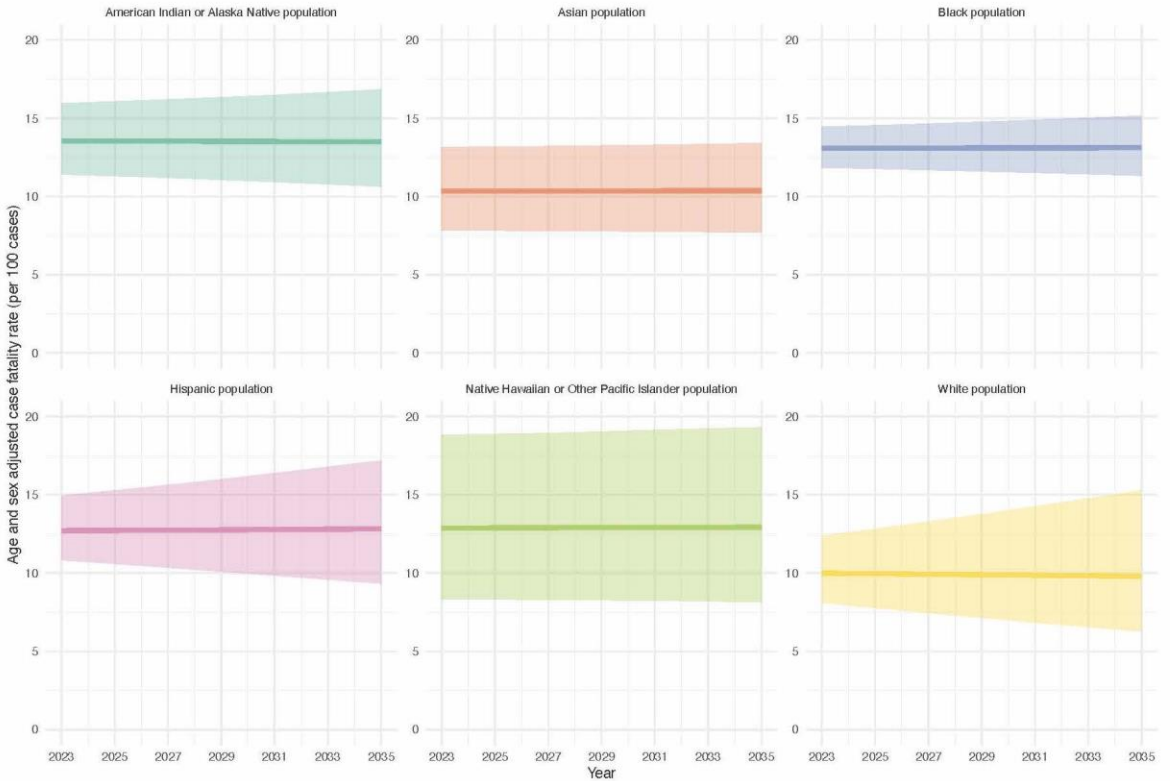

eFigure 4. Baseline Projections of Age- and Sex-Adjusted Rates Among US-Born Persons Between 2023 and 2035. Panel A: TB incidence rates (per 100 thousand persons) Panel B: TB case-fatality rates (per 100 cases)

© 2024 Swarwood NA et al. *JAMA Network Open.*

	Cases		Deaths		QALYs Lost	
	N	% of baseline	N	% of baseline	N	% of baseline
**Baseline estimates assuming 2010-2019 trends continue**						
Non-Hispanic American Indian or Alaska Native persons	1,035 (741–1,417)	100%	166 (140–193)	100%	2,452 (2,037–2,907)	100%
Non-Hispanic Asian persons	1,877 (1,349–2,562)	100%	86 (66–109)	100%	2,378 (1,835–3,015)	100%
Non-Hispanic Black persons	8,688 (7,804–9,652)	100%	1,254 (1,155–1,357)	100%	19,675 (17,795–21,759)	100%
Hispanic persons	6,643 (5,668–7,781)	100%	579 (474–694)	100%	12,101 (9,770–14,936)	100%
Non-Hispanic Native Hawaiian or Other Pacific Islander persons	711 (369 – 1,274)	100%	54 (34–79)	100%	1,345 (854–1,926)	100%
Non-Hispanic White persons	7,249 (6,444–8,138)	100%	1,125 (879–1,432)	100%	13,810 (10,442–17,822)	100%
**Total**	**26,203 (24,931–27,564)**	**100%**	**3,264 (3,051–3,513)**	**100%**	**51,761 (48,429–55,212)**	**100%**

© 2024 Swarwood NA et al. *JAMA Network Open.*

**Excess TB morbidity and mortality associated with racial and ethnic disparities only in TB case-fatality**						
Non-Hispanic American Indian or Alaska Native persons	720 (494–1,018)	69% (66–73)	119 (99–138)	71% (70–72)	2,290 (1,894–2,710)	94% (86–100%)
Non-Hispanic Asian persons	1,210 (820–1,724)	64% (60–68)	57 (43–72)	66% (65–66)	1,924 (1,470–2,473)	81% (69–95%)
Non-Hispanic Black persons	5,342 (4,724–6,022)	61% (60–63)	785 (716–858)	63% (62–63)	16,818 (15,191–18,609)	86% (79–92%)
Hispanic persons	4,089 (3,415–4,896)	62% (59–64)	365 (291–446)	63% (61–64)	9,777 (7,925–11,959)	81% (73–90%)
Non-Hispanic Native Hawaiian or Other Pacific Islander persons	540 (257–1,021)	75% (68–81)	42 (26–61)	78% (77–78)	1,213 (781–1,751)	90% (80–100%)
Non-Hispanic White persons	0	0%	0	0%	0	0%
**Total**	**11,901 (11,021 – 12,918)**	**45% (44–47)**	**1,368 (1,258 – 1,483)**	**42% (37–46)**	**32,022 (29,229–34,957)**	**62% (56 –67%)**
**Excess TB mortality associated with racial and ethnic disparities only in TB case-fatality**						

© 2024 Swarwood NA et al. *JAMA Network Open.*

Non-Hispanic American Indian or Alaska Native persons	Not applicable	Not applicable	33 (28–39)	20% (20–20)	861 (686–1,060)	35% (30–42%)
Non-Hispanic Asian persons	Not applicable	Not applicable	2 (2–3)	3% (2–3)	578 (343–880)	25% (14–38%)
Non-Hispanic Black persons	Not applicable	Not applicable	215 (197–235)	17% (17–17)	6,291 (5,306–7,329)	32% (27–38%)
Hispanic persons	Not applicable	Not applicable	96 (76–117)	17% (16–17)	3,859 (2,971–4,823)	32% (26–40%)
Non-Hispanic Native Hawaiian or Other Pacific Islander persons	Not applicable	Not applicable	10 (6–15)	19% (19–19)	471 (297–701)	35% (26–50%)
Non-Hispanic White persons	Not applicable	Not applicable	0	0%	0	0%
**Total**	Not applicable	Not applicable	**356 (328–387)**	**11% (10 –12)**	**12,060 (10,616–13,463)**	**23% (20–26%)**
**Excess TB morbidity and mortality associated with racial and ethnic disparities in TB incidence and case-fatality**						
Non-Hispanic American Indian or Alaska Native persons	720 (494–1,018)	70% (67–72)	121 (101–141)	73% (72–74)	2,329 (1,947–2,755)	95% (88–100%)

© 2024 Swarwood NA et al. *JAMA Network Open.*

Non-Hispanic Asian persons	1,210 (820–1,724)	64% (61–67)	57 (43–72)	66% (65–67)	1,931 (1,499–2,469)	81% (69–96%)
Non-Hispanic Black persons	5,342 (4,724–6,022)	61% (60–62)	817 (745–892)	65% (64–66)	17,394 (15,702–19,249)	88% (82–95%)
Hispanic persons	4,089 (3,415–4,896)	62% (60–63)	384 (306–469)	66% (64–68)	10,161 (8,214–12,401)	84% (75–93%)
Non-Hispanic Native Hawaiian or Other Pacific Islander persons	540 (257–1,021)	76% (70–80)	43 (27–63)	80% (79–80)	1,236 (779–1,791)	92% (82–100%)
Non-Hispanic White persons	0	0%	0	0%	0	0%
**Total**	**11,901 (11,021 – 12,918)**	**45% (44–47)**	**1,422 (1,307 – 1,543)**	**44% (39–48)**	**33,051 (30,181–36,277)**	**64% (58–70%)**

Values in parentheses represent 95% uncertainty intervals.

eTable 3. Estimated Baseline and Excess TB Morbidity and Mortality Associated With Racial and Ethnic Disparities in TB Incidence and Case-Fatality Among US-Born Persons, 2023 to 2035. Note: the non-Hispanic White population has already achieved the goal incidence rate and served as the referent for the goal case-fatality rate, there is no associated morbidity and mortality with this race-ethnicity.

© 2024 Swarwood NA et al. *JAMA Network Open.*

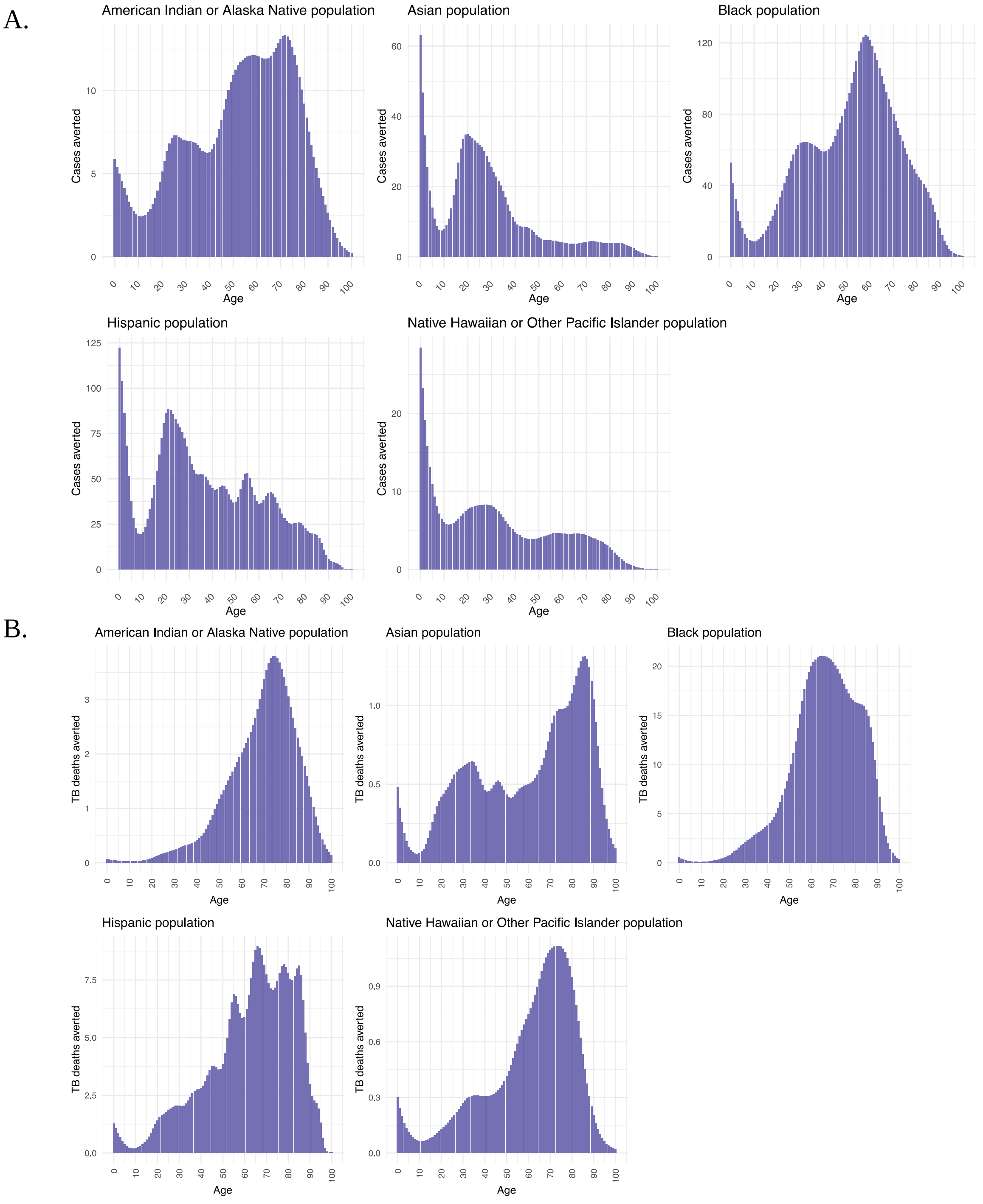

eFigure 5. Age Distribution of Cumulative TB Cases and Deaths Averted Among US-Born Persons During 2023 to 2035 Under the Both Disparities Removed Scenario for Each Race and Ethnicity.

Panel A: TB cases; Panel B: TB deaths.

© 2024 Swarwood NA et al. *JAMA Network Open.*

eResults

Additional baseline TB outcome estimates

The lowest estimated adjusted incidence rate (aIR) was among White persons, which ranged from 0.41 (0.36–0.45) in 2023 to 0.17 (0.14–0.20) per 100,000 in 2035**.** Estimated aIRs declined for all other persons, except among Native Hawaiian or Other Pacific Islander persons, for which the aIR was 3.76 (2.19–7.52) compared with 4.16 (1.73–11.41) per 100,000 between 2023 and 2035. Estimated aCFRs showed little change over all U.S.-born racial and ethnic populations during 2023–2035. The estimated QALYs lost per TB case was largest among American Indian or Alaska Native persons with 2.4 (1.8–3.1) QALYs lost per TB case and smallest among Asian persons with 1.3 (1.0–1.6) QALYs lost per TB case. Table S3 presents the distribution of cumulative QALYs lost to TB across each race and ethnicity.

---

[i] United States Mortality DataBase. Berkeley, CA (USA): University of California, Berkeley; 2020 [https://usa.mortality.org/, last accessed May 24 2021].
[ii] Guo N, Marra CA, Marra F, Moadebi S, Elwood RK, Fitzgerald JM. Health state utilities in latent and active TB. Value Health 2008; 11: 1154-1161.
[iii] Winston CA, Marks SM, Carr W. Estimated Costs of 4-Month Pulmonary Tuberculosis Treatment Regimen, United States. Emerging Infectious Diseases. 2023;29(10):2102-2104.
[iv] Jiao B, Basu A. U.S. Catalogue of Age- and Medical-Condition-Specific Healthcare Costs. 2021; https://uwchoice.shinyapps.io/futuremedicalcosts/. Accessed January 15, 2023.
[v] Taylor Z, Marks SM, Ríos Burrows NM, Weis SE, Stricof RL, Miller B. Causes and costs of hospitalization of tuberculosis patients in the United States. Int J Tuberc Lung Dis. 2000; 4:931– 939.
[vi] Shepardson D, Marks SM, Chesson H, Kerrigan A, Holland DP, Scott N, et al. Cost-effectiveness of a 12-dose regimen for treating latent tuberculous infection in the United States. Int J Tuberc Lung Dis 2013;17(12):1531-7.
[vii] Grosse SD, Krueger KV, Pike J. Estimated annual and lifetime labor productivity in the United States, 2016: implications for economic evaluations. *Journal of medical economics.* 2019;22(6):501-508.
[viii] U.S. Bureau of Labor Statistics. Table 1300. Age of reference person: Annual expenditure means, shares, standard errors, and coefficients of variation, Consumer Expenditure Surveys, 2021. 2022; https://www.bls.gov/cex/tables/calendar-year/mean-item-share-average-standard-error/reference-person-age-ranges-2021.pdf. Accessed January 15, 2023.

© 2024 Swarwood NA et al. *JAMA Network Open.*
