# Supplementary material for: Estimated Health and Economic Outcomes of Racial and Ethnic Tuberculosis Disparities in US-Born Persons
Source: JAMA Netw Open. 2024 Sep 10;7(9):e2431988. doi: 10.1001/jamanetworkopen.2024.31988 (PMC11388029; doi:10.1001/jamanetworkopen.2024.31988)
Supplement: Supplement 2. — Data Sharing Statement [file jamanetwopen-e2431988-s002.pdf]

Data Sharing Statement

Swartwood. Estimated Health and Economic Outcomes of Racial and Ethnic Tuberculosis Disparities in US-Born Persons. *JAMA Netw Open*. Published September 10, 2024. doi:10.1001/jamanetworkopen.2024.31988

Data
Data available: No

Additional Information
Explanation for why data not available: Apart from National TB Surveillance System data, all data used in this analysis represent deidentified publicly available datasets. National TB Surveillance System data contain information abstracted from the national tuberculosis case report form called the Report of Verified Case of Tuberculosis (RVCT) (OMB No. 0920-0728). These data have been reported voluntarily to CDC by state and local health departments and are protected under the Assurance of Confidentiality (Sections 306 and 308(d) of the Public Health Service Act, 42 U.S.C. 242k and 242m(d)), which prevents disclosure of any information that could be used to directly or indirectly identify patients. For more information, see the CDC/ATSDR Policy on Releasing and Sharing Data (at http://www.cdc.gov/maso/Policy/ReleasingData.pdf). A limited dataset is available at http://wonder.cdc.gov/tb.html. Researchers seeking additional National TB Surveillance System data may request access through the National Center for Health Statistics' Research Data Centers (https://www.cdc.gov/rdc/b1datatype/tuberculosis.htm). NAS had full access to all the data in the study and takes responsibility for the integrity of the data and the accuracy of the data analysis.
